# Changes in Colorectal Cancer Screening Modalities Among Insured Individuals

**DOI:** 10.1001/jamanetworkopen.2025.38578

**Published:** 2025-10-21

**Authors:** Sunny Siddique, Rong Wang, Folasade P. May, Laura V. M. Baum, Cary P. Gross, Xiaomei Ma

**Affiliations:** 1 Department of Chronic Disease Epidemiology, Yale School of Public Health, New Haven, Connecticut; 2 Division of Digestive Diseases, University of California Los Angeles Devid Geffen School of Medicine, Los Angeles; 3 Department of Medicine, Medical Oncology, Yale School of Medicine, New Haven, Connecticut; 4 Department of Medicine, General Medicine, Yale School of Medicine, New Haven, Connecticut

## Abstract

**Importance:**

Several colorectal cancer (CRC) screening modalities (eg, colonoscopy, stool-based tests) are currently available. Yet, recent national patterns in screening using these modalities and potential differences across sociodemographic groups remain unclear.

**Objective:**

To evaluate possible changes in the use of different CRC screening modalities among average-risk individuals aged 50 to 75 years in the US.

**Design, Setting, and Participants:**

This retrospective cohort study used deidentified claims data from 2017 to 2024. Participants included a dynamic cohort of commercially insured Blue Cross Blue Shield beneficiaries. Data were analyzed from May 1 to June 30, 2025.

**Exposures:**

Invasive and noninvasive CRC screening modalities.

**Main Outcomes and Measures:**

Changes in screening modality use were compared between the periods preceding the onset of the COVID-19 pandemic (ie, preonset: January 1, 2017, to February 28, 2020) and following the onset of the pandemic (ie, postonset: July 1, 2020, to December 31, 2024). Changes in screening by modality were also evaluated using autoregressive integrated moving average models, adjusting for temporal autocorrelation and seasonality.

**Results:**

In this retrospective cohort study of 24 973 642 distinct beneficiaries (mean [SD] age, 57.36 [4.27] years; 12 789 413 female [51.21%]), the use of colonoscopy (mean [SD], 1.29% [0.09%] to 1.14% [0.07%]; *P* < .001) and fecal immunochemical test (FIT) decreased between the 2 periods (mean [SD], 0.54% [0.03%] to 0.38% [0.07%]; *P* < .001). However, stool DNA test use increased (mean [SD], 0.19% [0.13%] to 0.61% [0.16%]; *P* < .001). Males had higher use of colonoscopy than females (mean [SD], 1.21% [0.07%] vs 1.07% [0.07%]; *P* < .001), whereas females had higher use of stool DNA test than males (mean [SD], 0.68% [0.16%] vs 0.55% [0.15%]; *P* < .001) and FIT (mean [SD], 0.43% [0.08%] vs 0.33% [0.05%]; *P* < .001). Areas with socioeconomic status (SES) in the top 20% had higher use of colonoscopy compared with areas where a marker of SES was in the bottom 20% (mean [SD], 1.37% [0.09%] vs 0.91% [0.06%]; *P* < .001) and stool DNA test (mean [SD], 0.65% [0.17%] vs 0.48% [0.13%]; *P* < .001) and lower use of FIT (mean [SD], 0.36% [0.09%] vs 0.48% [0.06%]; *P* < .001). Metropolitan area residents, compared with nonmetropolitan area residents, more frequently underwent colonoscopy (mean [SD], 1.18% [0.08%] vs 0.97% [0.06%]; *P* < .001) and FIT (mean [SD], 0.41% [0.08%] vs 0.25% [0.04%]; *P* < .001); however, their use of stool DNA test were similar (mean [SD], 0.61% [0.16%] vs 0.64% [0.16%]; *P* = .51).

**Conclusions and Relevance:**

Among privately insured individuals, the use of colonoscopy and FIT decreased after the COVID-19 pandemic while stool DNA test use increased, with differences by sex, area-level SES, and metropolitan area residence.

## Key Points

**Question:**

Did use of colorectal cancer (CRC) screening modalities change in the US between 2017 and 2024?

**Findings:**

In this cohort study of 24 973 642 privately insured individuals aged 50 to 75 years, the use of colonoscopy and fecal immunochemical test decreased, and stool DNA test use increased. Males had higher use of colonoscopy while females had higher use of stool DNA test; areas with high socioeconomic status (SES) and metropolitan areas had more frequent use of both modalities.

**Meaning:**

These findings suggest that use of different CRC screening modalities changed, varying by sex, area-level SES, and urban or rural residence.

## Teaser

This cohort study examines changes in the use of colorectal cancer screening modalities before and after the COVID-19 pandemic among average-risk individuals aged 50 to 70 years in the US.

## Introduction

Colorectal cancer (CRC) is the second leading cause of cancer death in the US.^1^ Over the past 2 decades, the incidence and mortality of CRC have decreased, largely attributed to the uptake of screening, which, on average, reduces CRC incidence by approximately 40% and mortality by about 60%.^2,3,4^ Despite these benefits, screening participation remains below the national target of 80% among adults aged 45 years and older, with particularly low screening observed among individuals aged 45 to 49 years, those identifying as Hispanic, Asian, American Indian, and Alaska Native, individuals born outside the US, and individuals with low income.^1,5^

Colonoscopy is the most commonly used, 1-step screening modality for CRC, combining CRC screening with the removal of precancerous adenomas for CRC prevention.^6^ Various other screening modalities are currently used based on individual risks, benefits, and preferences. These modalities range from stool-based tests (fecal occult blood tests [FOBT], fecal immunochemical tests [FIT], and multitarget stool DNA [stool DNA] test), to radiologic tests (computed tomographic [CT] colonography, double contrast barium enema [DCBE]), and other visual endoscopic examinations (flexible sigmoidoscopy). The novelty and heterogeneity of options, as well as the varying sensitivities and specificities of alternative modalities, in addition to the need for second-step colonoscopy following a positive screen on an alternative modality, may contribute to variable but low uptake of these screening tools. Generally, patient-level barriers and concerns (eg, fear of adverse events [eg, bleeding, perforation], cumbersome preparation process, need for anesthesia) and systems-level barriers (eg, high cost, need for time off work) may contribute to low colonoscopy screening rates in the general population.^7,8^ Additionally, the unique barriers to and preferences for colonoscopy and alternative screening modalities among specific demographic and socioeconomic subgroups may have further contributed to differences in screening.^9,10^

Several recent events have impacted the evolving landscape of CRC screening modalities. During the early phase of the COVID-19 pandemic (March to May 2020), colonoscopy was estimated to decrease by 85% in the US.^11^ Although this decline was potentially counterbalanced by an increase in stool-based testing at the peak of the pandemic, the extent to which alternative screening modalities were used after screening colonoscopies resumed remains unclear.^12^ Analysis of recent survey data has shown that among individuals aged 50 to 75 years, the CRC screening rate was approximately 61% in 2022.^13^ Yet, survey data are prone to response bias and often imputed for small geographic areas or areas with low population density. Therefore, a claims-based approach to evaluating CRC screening in the US is warranted.^14,15^ Furthermore, although insurance coverage has been identified as a key barrier to receiving CRC screening,^16^ use of various screening modalities among insured individuals and differences in screening test usage based on demographic and socioeconomic factors within this coverage group have not been evaluated in prior studies.

This study assessed recent patterns and potential shifts in the use of various CRC screening modalities among a large cohort of commercially insured individuals aged 50 to 75 years with average risk of CRC. Differences in the type of screening modality used were evaluated across groups with differing demographic and area-level socioeconomic characteristics while accounting for previously established disruptions to screening due to the COVID-19 pandemic. Individuals aged 50 to 75 years were chosen because screening recommendations for this age group remained the same during our study period (January 1, 2017, to December 31, 2024). Patterns in screening among individuals aged 45 to 49 years, who were recently recommended to receive CRC screening, have been reported previously.^17,18^ We focused on beneficiaries with average risk of CRC because the USPSTF screening recommendations apply to those at average risk; screening recommendations for those with high risk of CRC vary based on several factors, including individual and family history of colorectal polyps, inflammatory bowel disease, CRC, and other hereditary cancers.^19^

## Methods

### Data Source and Cohort Selection

The study population included individuals aged 50 to 75 years who are privately insured through Blue Cross Blue Shield (BCBS). BCBS is the largest provider of commercial insurance in the US, covering approximately one-third of the population. Over 92% of physicians and 96% of hospitals in the nation accept BCBS insurance, and BCBS is the only insurer data source that includes information from every zip code.^20^ The Yale University Institutional Review Board deemed our study to be nonhuman participants research, and therefore, we were exempt from seeking informed consent. We followed the Strengthening the Reporting of Observational Studies in Epidemiology (STROBE) reporting guideline.

We categorized the 96 months between January 1, 2017, and December 31, 2024 into 48 consecutive 2-month periods to capture potential shifts in screening patterns and short-term epidemiologic phenomena (eg, each wave of the COVID-19 pandemic) that may have impacted CRC screening. For each period, the denominator of our screening use calculation included average-risk beneficiaries who: (1) used BCBS as their primary insurance for at least 12 months prior to the 2-month period and remained enrolled through the end of the period and (2) did not have claims for any CRC screening related procedures in the 12 months preceding the period (eMethods and eTable 1 in Supplement 1). The numerator included the subset of the denominator who received CRC screening during the 2-month period of interest. Those who were screened more than once during the same period were counted as 1 individual in the numerator. Screening modality was assessed using claims for the following procedures: colonoscopy, CT colonography, flexible sigmoidoscopy, FOBT, FIT, stool DNA test, and DCBE (eMethods and eTable 1 in Supplement 1).^21,22,23,24^ To distinguish screening related procedures from diagnostic procedures, only outpatient procedures were included and, consistent with prior studies, those with claims for gastrointestinal tract symptoms, including abdominal pain, altered bowel habits, weight loss, iron deficiency anemia, fecal abnormalities, and gastrointestinal bleeding, within the 3 months preceding the period, were excluded.^25,26^ Since results of screening tests were not available, our analytic approach focused on test use in the periods of interest, rather than whether individuals were up to date with their recommended screening.

### Other Characteristics Evaluated

We used several individual and area-level measures of demographic and socioeconomic characteristics. Age and sex were known for all individuals in the study cohort, and imputed race and ethnicity information was available for approximately 80% of the cohort (eMethods in Supplement 1).^27^ Using beneficiaries’ zip code of residence, we calculated social deprivation index (SDI), a composite measure of area level deprivation based on 7 demographic characteristics collected in the American Community Survey.^28^ We also classified BCBS beneficiaries based on whether they resided in metropolitan or nonmetropolitan areas and their geographic regions.^29^ To do so, we used rural-urban commuting area codes that classify US zip codes with measures of population density, urbanization, and daily commuting.^30^

### Statistical Analysis

We compared bimonthly mean screening use by type of screening modality between the periods preceding (preonset: January 1, 2017, to February 28, 2020) and following (postonset: July 1, 2020, to December 31, 2024) the onset of the COVID-19 pandemic using 2-sided *t* tests (eMethods in Supplement 1). Differences in the use of screening based on demographic and socioeconomic attributes were evaluated using interrupted time-series analysis.^31^ The 4-month period between March 1 and June 30, 2020, was considered the interruption or break in the model. This method has been used previously to study the effect of large-scale policy interventions, including CRC screening and large-scale ecological phenomena.^18,32,33,34^

To calculate changes in screening use, we fit segmented regressions to the series of bimonthly screening uptake, with parameters for intercept, baseline trend, and changes in level and trend comparing the preonset and postonset periods. The Durbin-Watson statistic was used to check for autocorrelation, the tendency of adjacent time periods to share similar values.^31^ If autocorrelation was observed, we used autoregressive integrated moving average models that adjusted for autocorrelation as well as seasonality.^31,35,36^

All analyses were performed using R version 4.2.4 (R Project for Statistical Computing). All *P* values were based on 2-sided tests, and results were deemed statistically significant at *P* < .05. Data were examined from May 1 to June 30, 2025.

## Results

A total of 24 973 642 distinct beneficiaries aged 50 to 75 years (mean [SD] age, 57.36 [4.27] years; 12 789 413 female [51.21%]) with average risk of CRC were included in the study cohort. In this cohort, 22 903 327 individuals (91.71%) were aged 50 to 64 years and 2 070 315 (8.29%) were aged 65 to 75 years. Between January 1, 2017, and December 31, 2024, 7 686 887 beneficiaries (30.78%) received at least 1 type of CRC screening. Additionally, 10 433 987 beneficiaries resided in the southern census region of the US (41.78%), 5 416 782 resided in the midwestern region (21.69%), 4 218 048 resided in the northeastern region (16.89%), and 4 135 635 resided in the western region (16.56%). The most frequently used screening modalities were colonoscopy, stool DNA tests, FIT, and FOBT (Figure). The remaining screening modalities (eg, CT colonography, flexible sigmoidoscopy, and DCBE) accounted for less than 1% of screening tests completed during both periods.

**Figure.**
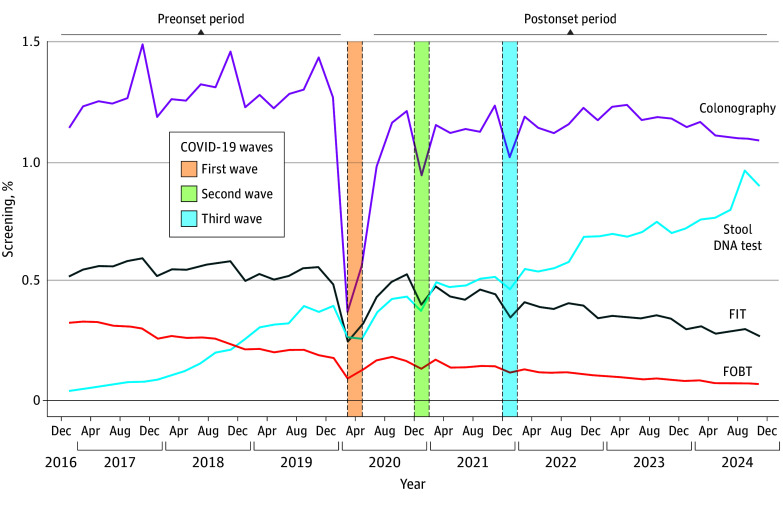
Bimonthly Colorectal Cancer Screening Use Among Blue Cross Blue Shield Beneficiaries Aged 50 to 75 Years FIT indicates fecal immunochemical test; FOBT, fecal occult blood test.

### Colonoscopy

Colonoscopy was the most frequently used screening modality in both the preonset and postonset periods (Figure). In the preonset period, a mean (SD) of 1.29% (0.09%) of the overall cohort received colonoscopy every 2 months. However, the mean (SD) colonoscopy use declined significantly in the postonset period to 1.14% (0.07%) (*P* < .001) (Table 1). Assessment of bimonthly screening rates showed no significant change in trend in colonoscopy use following the onset of the COVID-19 pandemic (increase of 0.002 [95% CI, −0.002 to 0.005] percentage point increase every 2 months).

**Table 1.** Changes in Bimonthly Colonoscopy Use Before and After the Onset of the COVID-19 Pandemic (January 2017 to February 2020 vs July 2020 to December 2024) Among Average-Risk Individuals Aged 50 to 75 Years and Insured by Blue Cross Blue Shield

Characteristic	Preonset			Postonset			*P* value comparing periods	Bimonthly percentage change (95% CI), %
	Frequency of beneficiaries screened, mean (SD)	CRC screening, mean (SD), %	*P* value comparing subgroups	Frequency of beneficiaries screened, mean (SD)	CRC screening, mean (SD), %	*P* value comparing subgroups		
Overall	115 437 (7306)	1.29 (0.09)	NA	89 824 (6281)	1.14 (0.07)	NA	<.001	0.002 (−0.002 to 0.005)
Sex								
Females	54 685 (4157)	1.20 (0.10)	<.001	43 190 (3135)	1.07 (0.08)	<.001	<.001	0.002 (−0.003 to 0.007)
Males	60 751 (3692)	1.38 (0.09)		46 634 (3359)	1.21 (0.07)		<.007	0.001 (−0.003 to 0.004)
Social deprivation index								
First (highest SES)	31 917 (2530)	1.51 (0.13)	NA	26 909 (1810)	1.37 (0.09)	NA	<.001	0.002 (−0.003 to 0.007)
Second	24 380 (1718)	1.31 (0.10)	<.001	19 826 (1196)	1.17 (0.08)	<.001	<.001	0.002(−0.002 to 0.006)
Third	20 507 (1322)	1.22 (0.08)	<.001	16 355 (969)	1.07 (0.07)	<.001	<.001	0.002 (−0.002 to 0.005)
Fourth	15 722 (977)	1.13 (0.07)	<.001	12 506 (724)	0.98 (0.06)	<.001	<.001	0.001 (−0.002 to 0.004)
Sixth (lowest SES)	9043 (414)	1.04 (0.05)	<.001	7510 (454)	0.91 (0.06)	<.001	<.001	0.002 (−0.001 to 0.004)
Locality								
Metropolitan	84 115 (5520)	1.32 (0.10)	<.001	70 161 (4265)	1.18 (0.08)	<.001	<.001	0.002 (−0.003 to 0.006)
Nonmetropolitan	18 448 (1380)	1.14 (0.08)		13 669 (813)	0.97 (0.06)		<.001	0.002 (−0.002 to 0.006)

Abbreviations: CRC, colorectal cancer; NA, not applicable; SES, socioeconomic status.

Males, compared with females, had higher use of colonoscopy in both the preonset period (mean [SD] bimonthly frequency, 60 751 [3 692] vs 54 685 [4157]; mean [SD], 1.38% [0.09%] vs 1.20% [0.10%]; *P* < .001) and the postonset period (mean [SD] bimonthly frequency, 46 634 [3 359] vs 43 190 [3135]; mean [SD], 1.21% [0.07%] vs 1.07% [0.08], *P* < .001). In the preonset period, beneficiaries residing in areas in the first SDI quintile (ie, highest socioeconomic status [SES]), had the highest use of colonoscopy, compared with beneficiaries residing in second to fifth SDI quintile areas (Table 1). Similarly, in the postonset period, first SDI quintile residents had higher use of colonoscopy, compared with second to fifth quintile residents (Table 1). Metropolitan area residents had higher use of colonoscopy compared with nonmetropolitan area residents in the preonset (mean [SD] bimonthly frequency, 84 115 [5520] vs 18 448 [1380]; mean [SD], 1.32% [0.10%] vs 1.14% [0.08%]; *P* < .001) and postonset (mean [SD] bimonthly frequency, 70 161 [4265] vs 13 669 [813]; mean [SD], 1.18% [0.08%] vs 0.97% [0.06%]; *P* < .001) periods.

### Stool DNA Test

In the preonset period, stool DNA tests were the fourth most frequently used screening modality after colonoscopy, FIT, and FOBT; however, in the postonset period, stool DNA tests were the second most common screening modality (Figure). The bimonthly stool DNA test use increased between the preonset and postonset periods (mean [SD], 0.19% [0.13%] to 0.61% [0.16%]; *P* < .001) (Table 2).

**Table 2.** Changes in Bimonthly Stool DNA Test Use Before and After the Onset of the COVID-19 Pandemic (January 2017 to February 2020 vs July 2020 to December 2024) Among Average-Risk Individuals Aged 50 to 75 Years and Insured by Blue Cross Blue Shield

Characteristic	Preonset			Postonset			*P* value comparing periods	Bimonthly percentage change (95% CI), %
	Frequency of beneficiaries screened, mean (SD)	CRC screening, mean (SD), %	*P* value comparing subgroups	Frequency of beneficiaries screened, mean (SD)	CRC screening, mean (SD), %	*P* value comparing subgroups		
Overall	16 598 (10 637)	0.19 (0.13)	NA	47 783 (10 063)	0.61 (0.16)	NA	<.001	0.028 (0.018 to 0.038)
Sex								
Females	9653 (6138)	0.22 (0.14)	.20	26 959 (5226)	0.68 (0.16)	.003	<.001	0.029 (0.017 to 0.041)
Males	6945 (4510)	0.16 (0.11)		20 824 (4867)	0.55 (0.15)		<.001	0.027 (0.017 to 0.036)
Social deprivation index								
First (highest SES)	4149 (2573)	0.20 (0.13)	NA	12 578 (2993)	0.65 (0.17)	NA	<.001	0.030 (0.019 to 0.041)
Second	3724 (2347)	0.21 (0.14)	.91	10 894 (2436)	0.65 (0.16)	.98	<.001	0.028 (0.017 to 0.040)
Third	3215 (2100)	0.20 (0.13)	.91	9536 (2216)	0.63 (0.16)	.71	<.001	0.029 (0.018 to 0.039)
Fourth	2471 (1647)	0.18 (0.13)	.64	7636 (1881)	0.60 (0.16)	.29	<.001	0.026 (0.017 to 0.353)
Fifth (lowest SES)	1208 (810)	0.14 (0.10)	.12	3990 (1046)	0.48 (0.13)	<.001	<.001	0.021 (0.012 to 0.029)
Locality								
Metropolitan	11 789 (7490)	0.19 (0.13)	.83	36 034 (8700)	0.61 (0.16)	.51	<.001	0.036 (0.021 to 0.051)
Nonmetropolitan	3115 (2066)	0.20 (0.14)		8970 (1940)	0.64 (0.16)		<.001	0.025 (0.011 to 0.038)

Abbreviations: CRC, colorectal cancer; NA, not applicable; SES, socioeconomic status.

Females, compared with males, had higher use of stool DNA tests in the preonset (mean [SD], 0.22% [0.14%] vs 0.16% [0.11%]; *P* = .20) and postonset (mean [SD], 0.68% [0.16%] vs 0.55% [0.15%]; *P* = .003) periods. In the preonset period, use of stool DNA tests was similar across areas with different SDI quintiles (Table 1). However, in the postonset period, use of stool DNA tests was significantly higher among first SDI quintile area residents compared with fifth SDI quintile residents (mean [SD], 0.65% [0.17%] vs 0.48% [0.13%]; *P* < .001). Use of stool DNA tests did not vary significantly between metropolitan and nonmetropolitan area residents in the preonset (mean [SD], 0.19% [0.13%] vs 0.20% [0.14%]; *P* = .83) and postonset (mean [SD], 0.61% [0.16%] vs 0.64% [0.16%]; *P* = .51) periods.

### FIT

In the preonset period, FIT was the second most frequently used screening modality after colonoscopy. However, in the postonset period, FIT was the third most frequently used modality after colonoscopy and stool DNA tests. The mean (SD) bimonthly FIT use decreased from 0.54% (0.03%) to 0.38% (0.07%) (*P* < .001) between the 2 periods (Table 3).

**Table 3.** Changes in Bimonthly Fecal Immunochemical Test Use Before and After the Onset of the COVID-19 Pandemic (January 2017 to February 2020 vs July 2020 to December 2024) Among Average-Risk Individuals Aged 50 to 75 Years and Insured by Blue Cross Blue Shield

Characteristic	Preonset			Postonset			*P* value comparing period	Bimonthly percentage change (95% CI), %
	Frequency of beneficiaries screened, mean (SD)	CRC screening, mean (SD), %	*P* value comparing subgroups	Frequency of beneficiaries screened, mean (SD)	CRC screening, mean (SD), %	*P* value comparing subgroups		
Overall	48 949 (3741)	0.54 (0.03)	NA	30 156 (6798)	0.38 (0.07)	NA	<.001	−0.008 (−0.010 to −0.007)
Sex								
Females	29 370 (2602)	0.64 (0.04)	<.001	17 535 (4220)	0.43 (0.08)	<.001	<.001	−0.011 (−0.012 to −0.009)
Males	19 579 (1259)	0.44 (0.02)		12 621 (2601)	0.33 (0.05)		<.001	−0.006 (−0.007 to −0.006)
Social deprivation index								
First (highest SES)	11 658 (1202)	0.55 (0.04)	NA	7153 (1865)	0.36 (0.09)	NA	<.001	−0.011 (−0.013 to −0.008)
Second	10 178 (876)	0.55 (0.04)	.82	6332 (1365)	0.37 (0.07)	.65	<.001	−0.009 (−0.010 to −0.007)
Third	8537 (683)	0.51 (0.03)	.001	5444 (1067)	0.36 (0.06)	.74	<.001	−0.008 (−0.009 to −0.006)
Fourth	7390 (474)	0.53 (0.03)	.08	4794 (812)	0.37 (0.06)	.60	<.001	−0.007 (−0.008 to −0.005)
Sixth (lowest SES)	5378 (247)	0.62 (0.03)	<.001	3940 (520)	0.48 (0.06)	<.001	<.001	−0.006 (−0.008 to −0.005)
Locality								
Metropolitan	37 629 (2823)	0.59 (0.03)	<.001	24 336 (4919)	0.41 (0.08)	<.001	<.001	−0.009 (−0.011 to −0.008)
Non-Metropolitan	5984 (559)	0.37 (0.02)		3604 (698)	0.25 (0.04)		<.001	−0.005 (−0.006 to −0.004)

Abbreviations: CRC, colorectal cancer; NA, not applicable; SES, socioeconomic status.

FIT was more frequently used among females, compared with males, in both the preonset (mean [SD], 0.64% [0.04%] vs 0.44% [0.02%]; *P* < .001) and postonset periods (mean [SD], 0.43% [0.08%] vs 0.33% [0.05%]; *P* < .001). FIT use was higher among beneficiaries residing in fifth SDI quintile areas, compared with first SDI quintile areas, in the preonset (mean [SD], 0.62% [0.03%] vs 0.55% [0.04%]; *P* < .001) and postonset periods (0.48% [0.06%] vs 0.36% [0.09%]; *P* < .001). Metropolitan area residents, compared with nonmetropolitan area residents, had higher use of FIT in the preonset (0.59% [0.03%] vs 0.38% [0.07%]; *P* < .001) and postonset periods (0.41% [0.08%] vs 0.25% [0.04%]; *P* < .001).

### FOBT

Between the preonset and postonset periods, FOBT use declined (0.26% [0.05%] to 0.11% [0.03%]; *P* < .001) (eResults and eTable 2 in Supplement 1). FOBT was more frequently used among females than males (0.13% [0.04%] vs 0.09% [0.03%]; *P* < .001), in higher SES areas (mean [SD], 0.13% [0.04%] in first SDI quintile areas vs 0.11% [0.03%] in the fifth SDI quintile areas; *P* < .001), and among metropolitan area residents (mean [SD], 0.12% [0.03%] in metropolitan areas vs 0.09% [0.03%] in nonmetropolitan area residents; *P* = .005) (Table 4).

**Table 4.** Changes in Bimonthly Fecal Occult Blood Test Use Before and After the Onset of the COVID-19 Pandemic (January 2017 to February 2020 vs July 2020 to December 2024) Among Average-Risk Individuals Aged 50 to 75 Years and Insured by Blue Cross Blue Shield

Characteristic	Preonset			Postonset			*P* value comparing periods	Bimonthly percentage change (95% CI), %
	Frequency of beneficiaries screened, mean (SD)	CRC screening, mean (SD), %	*P* value comparing subgroups	Frequency of beneficiaries screened, mean (SD)	CRC screening, mean (SD), %	*P* value comparing subgroups		
Overall	23 129 (5548)	0.26 (0.05)	NA	9109 (3105)	0.11 (0.03)	NA	<.001	−0.004 (−0.010 to 0.001)
Sex								
Females	12 827 (2852)	0.28 (0.05)	.007	5409 (1786)	0.13 (0.04)	<.001	<.001	−0.004 (−0.010 to 0.002)
Males	10 302 (2717)	0.23 (0.05)		3700 (1324)	0.09 (0.03)		<.001	−0.005 (−0.013 to 0.003)
Social deprivation index								
First (highest SES)	5952 (1412)	0.28 (0.05)	NA	2569 (828)	0.13 (0.04)	NA	<.001	−0.007 (−0.013 to −0.001)
Second	4757 (1212)	0.25 (0.05)	.15	1856 (597)	0.11 (0.03)	.03	<.001	−0.005 (−0.010 to 0.001)
Third	4086 (1005)	0.24 (0.05)	.03	1601 (528)	0.10 (0.03)	.009	<.001	−0.003 (−0.009 to 0.003)
Fourth	3466 (808)	0.25 (0.05)	.06	1401 (441)	0.11 (0.03)	.03	<.001	−0.004 (−0.010 to 0.001)
Fifth (lowest SES)	2195 (444)	0.25 (0.05)	.12	945 (265)	0.11 (0.03)	.09	<.001	−0.001 (−0.007 to 0.005)
Locality								
Metropolitan	17 073 (4002)	0.27 (0.05)	.008	7114 (2236)	0.12 (0.03)	.005	<.001	−0.004 (−0.010 to 0.001)
Nonmetropolitan	3610 (918)	0.22 (0.05)		1343 (445)	0.09 (0.03)		<.001	−0.002 (−0.008 to 0.003)

Abbreviations: CRC, colorectal cancer; NA, not applicable; SES, socioeconomic status.

### Exploratory and Sensitivity Analysis

Among the subset of beneficiaries with known race and ethnicity information, Hispanic individuals had the lowest bimonthly use of colonoscopy (0.87%), compared with non-Hispanic White individuals (1.20%) (*P* < .001) (eTable 2 in Supplement 1). However, Hispanic individuals had higher use of FIT (0.61%), compared with non-Hispanic White individuals (0.34%) (*P* < .001). Upon excluding those who had received FIT up to 9 months prior to the period of interest (instead of 12 months), we observed similar reduction in FIT use in the postonset period (0.68% vs 0.47%; *P* < .001) (eTable 3 in Supplement 1).

## Discussion

This large retrospective cohort study of 24 973 642 commercially insured individuals aged 50 to 75 years with average risk of CRC showed that the use of both colonoscopy and FIT decreased while stool DNA test use increased in the US since July 1, 2020. The use of colonoscopies was higher among males, while females had a higher use of stool-based tests. Except for FIT, use of each screening modality was higher among residents of high SES areas compared with low SES areas. Furthermore, residents of metropolitan areas experienced higher use of each screening modality compared with those in nonmetropolitan areas.

### Changes in Noninvasive Test Usage and Implications

Several recent studies have shown that among clinicians and gastroenterologists, colonoscopy is the preferred screening modality.^37,38^ This preference for colonoscopy is reflected in our cohort of BCBS beneficiaries, as colonoscopy was the most frequently used screening modality in both preonset and postonset periods. However, lower use of colonoscopy in the postonset period has been countered by increased use of stool DNA tests in high SES areas and FIT in low SES areas. The increase in stool DNA test use among BCBS beneficiaries may be due to multiple factors, including individual preferences and the success of advertising campaigns. Furthermore, the higher use of FIT in low SES areas may be attributed to its greater effectiveness and cost-effectiveness of FIT, as demonstrated in recent cost-effectiveness studies.^39,40^

### Sex Differences in Screening Modality and Implications

We observed a significant sex-based difference in the type of screening modality used among our study cohort. Colonoscopy use was higher among males, and stool-based test use was higher among females during both the preonset and postonset periods. Although it has been previously shown that CRC screening is higher overall among female BCBS beneficiaries, future research is needed to elucidate the lower use of colonoscopy among females.^41^ Few recent studies have evaluated sex-based differences in the type of CRC screening modality used and the unique barriers and facilitators to receiving CRC screening among males and females. One study in 2013 reported divergent themes associated with colonoscopy: bodily intrusion, perforation anxiety, and embarrassment among women and avoidant procrastination with underlying fatalism, concern for unnecessary health care, and uncomfortable vulnerability among men.^42^ Therefore, sex-specific screening promotion interventions may help increase screening among men and women who are at risk of low screening uptake.^43,44^ Furthermore, interventions designed to address multiple structural barriers, such as screening test outreach with as-needed patient navigation, may further increase screening.^45^

### SDI Differences in Screening Modality

We observed a higher use of colonoscopy, stool DNA test, and FOBT among residents in the first SDI quintile residents compared with second to fifth SDI quintile residents. However, FIT use was significantly higher among fifth SDI quintile residents compared with first SDI quintile residents in both the preonset and postonset periods. All beneficiaries in our study cohort had BCBS insurance, yet low use of colonoscopy and stool DNA tests in low SES areas may perpetuate previously observed screening differences.^41^ Future initiatives to increase the use of colonoscopy and stool DNA tests in low SES areas are needed to address these differences.

### Metropolitan and Nonmetropolitan Area Differences in Screening Modality and Implications

We observed higher use of colonoscopy, FIT, and FOBT among metropolitan area residents compared with nonmetropolitan area residents in both the preonset and postonset periods. One prior study showed that following the onset of the COVID-19 pandemic, overall screening differences between metropolitan and nonmetropolitan area residents were reduced.^41^ Our observation of similar stool DNA test usage between metropolitan and nonmetropolitan area residents may help explain the reduced differences that have been previously reported.

### Strengths and Limitations

Notable strengths of our study include longitudinal design, considerable sample size, national scale, and granular bimonthly screening data. Furthermore, our study calculated area-level measures using zip codes, which is smaller than county, hospital referral region, and state level data, and therefore, can capture small area-level variations in screening.

Nonetheless, our study has several limitations. Our study population may not be representative of the general US population because BCBS beneficiaries tend to be relatively younger and have employer-based insurance. Although BCBS covers every zip code of the US, the proportions of population covered by BCBS differed by states. Furthermore, the extent to which the cost of screening is covered may vary based on factors such as type of insurance, co-pay, and in-network status. However, prior studies have shown that cancer care patterns among BCBS beneficiaries are similar to those of Medicare beneficiaries.^46^ Since test results were not available, we were unable to assess whether the beneficiaries were up to date with their recommended screening. Yet, our exploratory analysis showed that approximately 30.78% of beneficiaries in this cohort received at least 1 type of CRC screening during the study period. This estimate may be lower than the national estimate of 60% to 69% of the US population being up to date with their recommended screening because our study cohort was observed for a relatively short time period. Furthermore, we did not require our beneficiaries to be continually enrolled in BCBS throughout the study period. Therefore, screening received outside of BCBS insurance, if any, would not have been captured in our study. Lastly, the results of this study do not account for potential geographic differences in screening preference and variations in local and state-level restrictions that delayed elective procedures, such as screening colonoscopies.

## Conclusions

In this retrospective cohort study of commercially insured individuals in the US, the use of colonoscopy and FIT decreased while stool DNA test use increased. Males were more likely to use colonoscopy, whereas females were more likely to use stool-based tests. Areas with high SES and metropolitan area residents had higher use of colonoscopy and stool DNA tests, although use of FIT was higher among low SES areas. The heterogeneity in screening modality use based on population subgroups warrants tailored interventions to increase screening participation for all.

## Notes

Supplement 1.
eMethods.
**eTable 1. **Codes used to identify colorectal cancer screening related procedures and high-risk individuals **eTable 2. **Changes in bimonthly colorectal cancer screening before and after the onset of the COVID-19 pandemic (January 2017 to February 2020 vs July 2020 to December 2024) Among average-risk individuals aged 50 to 75 years and insured by Blue Cross Blue Shield stratified by race and ethnicity **eTable 3. **Percentage of BCBS beneficiaries who were aged 50 to 75 years between June 2020 to December 2024 and received any screening for colorectal cancer **eTable 4. **Changes in bimonthly colorectal cancer screening before and after the onset of the COVID-19 pandemic (January 2017 to February 2020 vs July 2020 to December 2024) among average-risk individuals aged 50 to 75 years and insured by Blue Cross Blue Shield
eReferences

Supplement 2.
Data Sharing Statement
