# Supplementary material for: Changes in Colorectal Cancer Screening Modalities Among Insured Individuals
Source: JAMA Netw Open. 2025 Oct 21;8(10):e2538578. doi: 10.1001/jamanetworkopen.2025.38578 (PMC12541536; doi:10.1001/jamanetworkopen.2025.38578)
Supplement: Supplement 1. — eMethods. eTable 1. Codes used to identify colorectal cancer screening related procedures and high-risk individuals eTable 2. Changes in bimonthly colorectal cancer screening before and after the onset of the COVID-19 pandemic (January 2017 to February 2020 vs July 2020 to December 2024) Among average-risk individuals aged 50 to 75 years and insured by Blue Cross Blue Shield stratified by race and ethnicity eTable 3. Percentage of BCBS beneficiaries who were aged 50 to 75 years between June 2020 to December 2024 and received any screening for colorectal cancer eTable 4. Changes in bimonthly colorectal cancer screening before and after the onset of the COVID-19 pandemic (January 2017 to February 2020 vs July 2020 to December 2024) among average-risk individuals aged 50 to 75 years and insured by Blue Cross Blue Shield eReferences [file jamanetwopen-e2538578-s001.pdf]

Supplemental Online Content

Siddique S, Wang R, May FP, et al. Changes in colorectal cancer screening modalities among insured individuals in the US. *JAMA Netw Open*. 2025;8(10):e2538578.
doi:10.1001/jamanetworkopen.2025.38578

eMethods.

eTable 1. Codes used to identify colorectal cancer screening related procedures and high-risk individuals

eTable 2. Changes in bimonthly colorectal cancer screening before and after the onset of the COVID-19 pandemic (January 2017 to February 2020 vs July 2020 to December 2024) Among average-risk individuals aged 50 to 75 years and insured by Blue Cross Blue Shield stratified by race and ethnicity

eTable 3. Percentage of BCBS beneficiaries who were aged 50 to 75 years between June 2020 to December 2024 and received any screening for colorectal cancer

eTable 4. Changes in bimonthly colorectal cancer screening before and after the onset of the COVID-19 pandemic (January 2017 to February 2020 vs July 2020 to December 2024) among average-risk individuals aged 50 to 75 years and insured by Blue Cross Blue Shield

eReferences

This supplemental material has been provided by the authors to give readers additional information about their work.

© 2025 Siddique S et al. *JAMA Network Open*.

eMethods

Determination of Risk Status

Since CRC screening recommendations are applicable to individuals with “average-risk” of CRC, we identified the risk status of our beneficiaries. High-risk individuals were identified using the following criteria: history of colorectal polyps, CRC, irritable bowel disease, family history of gastrointestinal cancer, and genetic susceptibility to other malignant neoplasms.[1] Individuals without a history of these conditions were classified as having “average-risk” of CRC.

Screening vs Diagnostic Procedures

Screening related procedures were distinguished from diagnostic procedures by including only outpatient procedures. To distinguish screening related procedures from diagnostic procedures, only outpatient procedures were included and those with claims for gastrointestinal tract symptoms, including abdominal pain, altered bowel habits, weight loss, iron deficiency anemia, fecal abnormalities, and gastrointestinal bleeding, within the 3 months preceding the period, were excluded.[2,3] Individuals diagnosed with CRC based on diagnosis code were censored before the month in which the first CRC-related code appeared.

Events of Interest

Several pandemic-related events occurred during the study period: 1) first wave of the COVID outbreak (March 1, 2020 to April 30, 2020); 2) second wave of the COVID outbreak (January 1, 2021 to February 28, 2021); 3) third wave of the COVID outbreak (January 1, 2022 to March 31, 2022).[4-6] Upon consideration of these events, we compared changes in screening modalities during the period preceding (January 1, 2017 to February 28, 2020) and following (July 1, 2021 to December 31, 2022) the onset of the COVID pandemic. The period between March 1, 2020 to June 30, 2020 was not included in our analysis to allow for a brief “washout” period at the peak of the pandemic when CRC screening was suspended across the US.[7]

© 2025 Siddique S et al. *JAMA Network Open*.

eTable 1. Codes used to identify colorectal cancer screening related procedures and high-risk individuals

Codes used to identify colorectal cancer screening		
Procedure Name	Code Type	Code
Fecal occult blood test	CPT/HCPCS	G0107, 82270, 82271, 82272, 82273
Fecal immunochemical test	CPT/HCPCS	G0328, 82274
Stool DNA test (Cologuard®)	CPT/HCPCS	81528, G0464 , S3890 ALSO BY NPI
Flexible sigmoidoscopy	CPT/HCPCS	G0104, G6022, 45300, 45303, 45305, 45307, 45308, 45309, 45315, 45317, 45320, 45321, 45327, 45330, 45331, 45332, 45333, 45334, 45335, 45337,45338, 45339, 45340, 45341, 45342, 45345, 45346, 45349
Double-contrast barium enema	CPT/HCPCS	G0106, G0120, G0122, 74270, 74280
Colonoscopy	CPT/HCPCS	G0105, G0121, G6024, 44388-44394, 44397, 45355, 45378, 45379, 45380, 45381, 45382, 45383, 45384, 45385, 45386, 45387, 45388, 45390, 45391, 45392
CT colonography	CPT/HCPCS	74263, 0066T
**Codes used to identify high risk individuals**		
High-risk Condition	Code Type	Codes
Colorectal polyp	ICD-9-CM	2113, 2114, 5690, 5564, 56949, 20950–20957
	ICD-10-CM	D120–D128, K5140, K51411–K51414, K51418, K51419, K620, K621, K635, D3A020–D3A026, D3A029
History of colorectal polyp	ICD-9-CM	V1272
	ICD-10-CM	Z86010
Colorectal cancer	ICD-9-CM	1530–1541, 1548, 1975, 2303, 2304, 20910–20917
	ICD-10-CM	C180–C189, C19, C20, C212, C218, C785, C7A020–C7A026, C7A029, D0140, D0149, D010–D012
History of colorectal cancer	ICD-9-CM	V1005, V1006
	ICD-10-CM	Z85030, Z85038, Z85040, Z85048, Z8509
Family history of gastrointestinal cancer	ICD-9-CM	V160
	ICD-10-CM	Z800

© 2025 Siddique S et al. *JAMA Network Open*.

Irritable bowel disease	ICD-9-CM	5550–5552, 5559–5563, 5565, 5566, 5568, 5569
	ICD-10-CM	K5000, K50011–K50014, K50018, K50019, K5010, K5011, K50111–K50114, K50118, K50119, K508, K5080, K50811–K50814, K50818, K50819, K5090, K50911–K50914, K50918, K50919, K5100, K51011–K51014, K51018, K51019, K5120, K51211–K51214, K51218, K51219, K513, K5130, K51311–K51314, K51318, K51319, K5150, K51511–K51514, K51518, K51519, K5180, K51811–K51814, K51818, K51819, K5190, K51911–K51914, K51918, K51919, K5289
Genetic susceptibility to other malignant neoplasm	ICD-9-CM	V8409
	ICD-10-CM	Z1509

Codes used to distinguish diagnostic procedures from screening related procedures

Condition	Code Type	Code
Abdominal Pain	ICD-9-CM	787.3, 789.6, 789
	ICD-10-CM	R14, R10.81, R10.82
Altered bowel habits	ICD-9-CM	564,787
	ICD-10-CM	K52.29, K52.89, K58, K59 EXCEPT K59.31, R11.0, R11.10, R11.11-2, R11.14-5, R12-5, R19.11-12, R19.15, R19.4-5, R19.7, R19.8
Gastrointestinal bleeding	ICD-9-CM	578
	ICD-10-CM	K92.0-2
Fecal abnormalities	ICD-9-CM	792.1
	ICD-10-CM	R19.5
Weight loss	ICD-9-CM	783.2
	ICD-10-CM	R63.4, R63.6
Iron deficiency anemia	ICD-9-CM	280
	ICD-10-CM	D50
Anemia unspecified	ICD-9-CM	285.9
	ICD-10-CM	D64.9
Sigmoidoscopy	ICD-9-CM	45.24, 48.21-4
	ICD-10-CM	0DJD8ZZ, 0D9P3ZX

© 2025 Siddique S et al. *JAMA Network Open*.

eTable 2. Changes in bimonthly colorectal cancer screening before and after the onset of the COVID-19 pandemic (January 2017 to February 2020 vs July 2020 to December 2024) among average-risk individuals aged 50 to 75 years and insured by Blue Cross Blue Shield stratified by race and ethnicity [a]

Colonoscopy

	Pre-Onset			Post-Onset			P-value Comparing Pre- vs Post-Onset	Bimonthly Percentage Change (%, 95% CI)
	Mean Frequency of Beneficiaries Screened (SD)	Mean CRC Screening (%, SD)	P-value Comparing Subgroups	Mean Frequency of Beneficiaries Screened (SD)	Mean CRC Screening (%, SD)	P-value Comparing Subgroups		
**Race and Ethnicity**								
Hispanic	4,870 (602)	0.98 (0.05)	<0.001	5,266 (531)	0.87 (0.08)	<0.001	<0.001	0.002 (0.002, 0.008)
NH API	3,068 (472)	1.20 (0.14)	0.87	3,365 (493)	1.13 (0.17)	0.06	<0.001	0.016 (0.005, 0.027)
NH Black	6,217 (387)	1.16 (0.06)	0.02	5,588 (426)	1.05 (0.07)	<0.001	<0.001	0.002 (-0.006, 0.000)
NH White	81,780 (5,534)	1.35 (0.09)	Ref	66,180 (5,160)	1.20 (0.07)	Ref	<0.001	0.001 (-0.003, 0.005)
Unknown	18,488 (6,818)	1.19 (0.10)	0.88	8,383 (598)	1.02 (0.07)	<0.001	<0.001	0.002 (-0.002, 0.005)

Stool DNA Test

	Pre-Onset			Post-Onset			P-value Comparing Pre- vs Post-Onset	Bimonthly Percentage Change (%)
	Mean Frequency of Beneficiaries Screened (SD)	Mean CRC Screening (%, SD)	P-value Comparing Subgroups	Mean Frequency of Beneficiaries Screened (SD)	Mean CRC Screening (%, SD)	P-value Comparing Subgroups		
**Race and Ethnicity**								
Hispanic	662 (539)	0.12 (0.09)	0.04	2,893 (822)	0.48 (0.12)	<0.001	<0.001	0.019 (0.010, 0027)
NH API	325 (260)	0.12 (0.09)	<0.001	1,554 (543)	0.52 (0.18)	0.01	<0.001	0.025 (0.017, 0.034)
NH Black	696 (513)	0.13 (0.09)	0.05	2,522 (742)	0.48 (0.15)	<0.001	<0.001	0.029 (0.020, 0.039)
NH White	12,609 (8,482)	0.21 (0.14)	Ref	35,729 (6,917)	0.66 (0.16)	Ref	<0.001	0.031 (0.020, 0.042)

© 2025 Siddique S et al. *JAMA Network Open*.

Unknown	2,151 (754)	0.17 (0.11)	0.43	4,499 (954)	0.55 (0.14)	<0.001	<0.001	0.028 (0.019, 0.036)

Fecal Immunochemical Test (FIT)

	Pre-Onset			Post-Onset			P-value Comparing Pre- vs Post-Onset	Bimonthly Percentage Change (%)
	Mean Frequency of Beneficiaries Screened (SD)	Mean CRC Screening (%, SD)	P-value Comparing Subgroups	Mean Frequency of Beneficiaries Screened (SD)	Mean CRC Screening (%, SD)	P-value Comparing Subgroups		
**Race and Ethnicity**								
Hispanic	4,123 (684)	0.82 (0.06)	<0.001	3,655 (382)	0.61 (0.08)	<0.001	<0.001	-0.006 (-0.010, -0.002)
NH API	2,373 (384)	0.91 (0.11)	<0.001	2,381 (397)	0.80 (0.14)	<0.001	<0.001	-0.009 (-0.012, -0.002)
NH Black	2,824 (120)	0.53 (0.03)	0.05	2,087 (44)	0.39 (0.08)	0.01	<0.001	-0.009 (-0.010, -0.007)
NH White	30,867 (1,590)	0.51 (0.03)	Ref	18,733 (5,070)	0.34 (0.07)	Ref	<0.001	-0.010 (-0.011, -0.007)
Unknown	8,151 (3,429)	0.51 (0.04)	0.9	2,807 (629)	0.34 (0.06)	0.02	<0.001	-0.002 (-0.011, -0.005)

Fecal Occult Blood Test

	Pre-Onset			Post-Onset			P-value Comparing Pre- vs Post-Onset	Bimonthly Percentage Change (%)
	Mean Frequency of Beneficiaries Screened (SD)	Mean CRC Screening (%, SD)	P-value Comparing Subgroups	Mean Frequency of Beneficiaries Screened (SD)	Mean CRC Screening (%, SD)	P-value Comparing Subgroups		
**Race and Ethnicity**								
Hispanic	1,239 (116)	0.25 (0.05)	0.87	680 (163)	0.11 (0.03)	0.91	<0.001	-0.005 (-0.010, -0.001)
NH API	763 (101)	0.30 (0.06)	0.02	380 (102)	0.13 (0.03)	0.15	<0.001	0.000 (-0.009, 0.010)
NH Black	1,373 (204)	0.26 (0.05)	0.88	643 (221)	0.12 (0.04)	0.56	<0.001	-0.006 (-0.011, -0.001)
NH White	15,460 (2,922)	0.26 (0.05)	Ref	6,418 (2,279)	0.11 (0.03)	Ref	<0.001	-0.004 (-0.010, 0.001)
Unknown	40,59 (2,285)	0.24 (0.05)	0.42	875 (322)	0.11 (0.03)	0.27	<0.001	-0.004 (-0.009, 0.000)

Abbreviations: CRC = Colorectal Cancer, NH = non-Hispanic, SD = Standard Deviation, SES = Socioeconomic Status, API = Asian/Pacific Islander.

© 2025 Siddique S et al. *JAMA Network Open*.

[a] Race and ethnicity information was available for approximately 80% of the overall population

© 2025 Siddique S et al. *JAMA Network Open*.

eTable 3. Percentage of BCBS beneficiaries who were aged 50 to 75 years and received any screening during 2017 to 2024

	Sample size	Ever Screened	Screening Uptake (%)
Ever average risk [a]	24,973,642	8,376,730	33.54%
Stayed average risk [b]	20,225,606	4,959,983	24.52%
Transitioned from average risk to high risk [c]	4,748,036	3,416,747	71.96%
Stayed high risk [d]	2,961,583	946,691	31.97%

[a] Includes individuals aged 50-75 years who were ever in "average risk" of CRC had a screening before age 50.

[b] Includes individuals aged 50-75 years who stayed "average risk" during the study period.

[c] Includes individuals aged 50-75 years who transitioned from "average risk" to "high risk" during the study period.

[d] Includes individuals aged 50-75 years who stayed "high risk" during the study period.

© 2025 Siddique S et al. *JAMA Network Open*.

eTable 4. Changes in bimonthly colorectal cancer screening before and after the onset of the COVID-19 pandemic (January 2017 to February 2020 vs July 2020 to December 2024) among average-risk individuals aged 50 to 75 years and insured by Blue Cross Blue Shield

Fecal Immunochemical Test (FIT) (9 month cutoff)								
	Pre-Onset			Post-Onset			P-value Comparing Pre- vs Post-Onset	Bimonthly Percentage Change (%)
	Mean Frequency of Beneficiaries Screened (SD)	Mean CRC Screening (%, SD)	P-value Comparing Subgroups	Mean Frequency of Beneficiaries Screened (SD)	Mean CRC Screening (%, SD)	P-value Comparing Subgroups		
**Overall**	62,120 (5,248)	0.68 (0.04)		37,412 (7,922)	0.47 (0.079)		<0.001	-0.009 (-0.011, -0.007)
**Sex**								
Females	29,370 (2,602)	0.64 (0.04)	<0.001	17,535 (4,220)	0.43 (0.08)	<0.001	<0.001	-0.001 (-0.007, -0.005
Males	19,579 (1,259)	0.44 (0.02)		12,621 (2,601)	0.33 (0.05)		<0.001	-0.007 (-0.008, -0.006)
**Social Deprivation Index**								
1st (highest SES)	15,196 (1,672)	0.71 (0.06)	Ref	9,137 (2,226)	0.46 (0.10)	Ref	<0.001	-0.012 (-0.016, -0.009)
2nd	13,004 (1,204)	0.69 (0.05)	0.004	7,952 (1,626)	0.46 (0.08)	0.87	<0.001	-0.001 (-0.012, -0.008)
3rd	10,797 (930)	0.64 (0.04)	<0.001	6,744 (1,232)	0.44 (0.07)	0.35	<0.001	-0.008 (-0.011, -0.006)
4th	9,274 (5,872)	0.66 (0.04)	0.003	5,872 (909)	0.45 (0.06)	0.78	<0.001	-0.007 (-0.009, -0.056)
5th (lowest SES)	6,577 (322)	0.75 (0.04)	0.014	4,704 (570)	0.56 (0.07)	<0.001	<0.001	-0.007 (-0.009, -0.005)
**Locality**								
Metropolitan	47,905 (4,006)	0.74 (0.05)	<0.001	30,294 (5,737)	0.50 (0.09)	<0.001	<0.001	-0.010 (-0.012, -0.008)
Non-Metropolitan	7,541 (740)	0.46 (0.03)		4,463 (809)	0.31 (0.05)		<0.001	-0.006 (-0.007, -0.004)
**Race and Ethnicity [a]**								
Hispanic	4,788 (792)	0.94 (0.07)	<0.001	4,195 (405)	0.69 (0.08)	<0.001	<0.001	-0.006 (-0.010, -0.001)
NH API	2,985 (494)	1.15 (0.14)	0.03	2,950 (473)	0.98 (0.16)	<0.001	<0.001	-0.007 (-0.017, 0.002)

© 2025 Siddique S et al. *JAMA Network Open*.

NH Black	3,565 (163)	0.66 (0.04)	0.54	2,562 (498)	0.48 (0.08)	0.02	<0.001	-0.010 (-0.012, -0.008)
NH White	39,875 (2,411)	0.65 (0.05)	Ref	23,665 (6,009)	0.42 (0.08)	Ref	<0.001	-0.011 (-0.013, -0.008)
Unknown	10,163 (4,294)	0.63 (0.06)	0.22	3,448 (728)	0.41 (0.06)	0.67	<0.001	-0.009 (-0.012, -0.005)

Abbreviations: CRC = Colorectal Cancer, NH = non-Hispanic, SD = Standard Deviation, SES = Socioeconomic Status, API = Asian/Pacific Islander.

[a] Race and ethnicity information was available for approximately 80% of the overall population

© 2025 Siddique S et al. *JAMA Network Open*.

eReferences

1. Engel-Nitz NM, Miller-Wilson LA, Le L, Limburg P, Fisher DA. Colorectal screening among average risk individuals in the United States, 2015-2018. *Prev Med Rep.* 2023;31:102082.
2. Khiani VS, Soulos P, Gancayco J, Gross CP. Anesthesiologist involvement in screening colonoscopy: temporal trends and cost implications in the medicare population. *Clin Gastroenterol Hepatol.* 2012;10(1):58-64 e51.
3. Ko CW, Kreuter W, Baldwin LM. Effect of Medicare coverage on use of invasive colorectal cancer screening tests. *Arch Intern Med.* 2002;162(22):2581-2586.
4. Fedewa SA, Star J, Bandi P, et al. Changes in Cancer Screening in the US During the COVID-19 Pandemic. *JAMA Netw Open.* 2022;5(6):e2215490.
5. Mazidimoradi A, Tiznobaik A, Salehiniya H. Impact of the COVID-19 Pandemic on Colorectal Cancer Screening: a Systematic Review. *J Gastrointest Cancer.* 2022;53(3):730-744.
6. El-Shabasy RM, Nayel MA, Taher MM, Abdelmonem R, Shoueir KR, Kenawy ER. Three waves changes, new variant strains, and vaccination effect against COVID-19 pandemic. *Int J Biol Macromol.* 2022;204:161-168.
7. Ross JS, Wang R, Long JB, Gross CP, Ma X. Impact of the 2008 US Preventive Services Task Force recommendation to discontinue prostate cancer screening among male Medicare beneficiaries. *Arch Intern Med.* 2012;172(20):1601-1603.

© 2025 Siddique S et al. *JAMA Network Open*.
