# Supplementary material for: Changes in Colorectal Cancer Screening Modalities Among Insured Individuals
Source: JAMA Netw Open. 2025 Oct 21;8(10):e2538578. doi: 10.1001/jamanetworkopen.2025.38578 (PMC12541536; doi:10.1001/jamanetworkopen.2025.38578)
Supplement: Supplement 2. — Data Sharing Statement [file jamanetwopen-e2538578-s002.pdf]

Data Sharing Statement

Data
Data available: No

Additional Information
Explanation for why data not available: We obtained access to the BCBS Axis data through an institutional agreement on research collaboration between Yale University and BCBS and a proposal describing the work presented in this manuscript that was approved by the BCBS. We are not allowed to share the raw data as stipulated by the institutional agreement and the proposal. Interested investigators should contact BCBS directly for potential data access. On the other hand, we are open to sharing our analytical methods and statistical programs with any researchers who contact us.
